# Critical Mentorship in Undergraduate Research Experience BUILDs Science Identity and Self-Efficacy

**DOI:** 10.1007/s10763-024-10476-0

**Published:** 2024-06-20

**Authors:** Sungmin Moon, Shu-Sha Angie Guan, Jose H. Vargas, Judith C. P. Lin, Patchareeya Kwan, Carrie L. Saetermoe, Gilberto Flores, Gabriela Chavira

**Affiliations:** 1 Department of Psychology, California State University Northridge, Northridge, CA 91330 USA; 2 Department of Child and Adolescent Development, California State University Northridge, Northridge, CA 91330 USA; 3 Health Equity Research and Education Center, California State University Northridge, Northridge, CA 91330 USA; 4 Department of Health Sciences, California State University Northridge, Northridge, CA 91330 USA; 5 Department of Biology, California State University Northridge, Northridge, CA 91330 USA

**Keywords:** Critical Race Theory (CRT), Critical mentoring, Science identity, Science self-efficacy

## Abstract

In 2014, the NIH Diversity Program Consortium (DPC) launched an initiative to implement and evaluate novel interventions at a variety of academic institutions across the country to engage undergraduate students from diverse backgrounds in biomedically-related research. The local intervention examined in the current study provides Critical Race Theory (CRT)-informed mentoring, more broadly called critical mentoring, for its participants. We examined the relationship between critical mentoring and student outcomes. In this study, student outcomes consisted of three components: (a) mentor satisfaction, (b) science identity, and (c) science self-efficacy. To determine student outcomes, we used the 2020 Student Annual Follow-up Survey (SAFS). We found that participants in the intervention program reported higher levels of critical mentoring than non-intervention participants and critical mentoring was, in turn, predictive of higher. mentorship satisfaction, science identity, and science self-efficacy. This finding implies that the CRT-informed intervention was more effective by developing an environment in which high-quality, critical mentors influenced students’ sense of science identity and self-efficacy. Additionally, we also found that intervention participants reported higher science identity and science self-efficacy than non-intervention participants, which suggests that the intervention cultivated science identity and self-efficacy in other ways outside of critical mentorship as well. The current study highlights how participation in an intervention program can increase science identity and self-efficacy, two factors predictive of science career intentions. The connection between critical mentoring practices and increased science identity and self-efficacy underscores the significance of culturally and racially relevant social support in science education.

## Diversity in STEM

Conditions imposed by structural racism continue to constrain representation and social justice in science, technology, engineering, and mathematics (STEM) disciplines. Data consistently reveal that traditionally underserved racial/ethnic group (URG) students are less likely to major and persist in STEM than their white male counterparts (Anfuso et al., 2022; Eagan et al., 2013; Flynn, 2016; Griffiths, 2010). Similarly, URG students earn college STEM degrees at relatively lower rates (NSF, 2017; U.S. Department of Education, 2020a, b). Selective student pushout feeds institutional-level factors such as a lack of diverse STEM faculty and contributes to representational inequities in mentor access among URG students (Basile & Murray, 2015). While URG students make up about 37% of the overall U.S. resident undergraduate student population in fall 2020 (National Center for Education Statistics, 2022), comparable research shows that URG faculty represent only one-tenth of the tenured faculty population (U.S. Department of Education, 2020c). Further, URG students earn 21% of STEM Bachelor’s degrees in 2020–21 (National Center for Education Statistics, 2023), yet only about 9% of STEM academic faculty are from URG backgrounds (NSF NCSES, 2019).

Underrepresentation of faculty of color limits opportunities for URG students to meet and learn from faculty with whom they share culturally and experientially relevant identities, backgrounds, and social justice challenges. This is problematic because URG students often benefit academically when mentored by URG faculty (Campbell & Campbell, 2007; Jaeger et al., 2017; Kricorian et al., 2020; Pope, 2002; Quaye et al., 2015). Additional advantages associated with STEM diversity include enhanced STEM interest, critical thinking, and self-efficacy among URG students (Hall et al., 2017; Strayhorn, 2009), improved job satisfaction and perceived treatment among URG faculty (Smith et al., 2017), and economic/cultural gains for universities, the STEM field, and society more broadly (Tremaine et al., 2022).

Critical and antiracist perspectives in undergraduate research experiences (UREs) can disrupt the cycle of inequity and shift academic paradigms toward STEM diversification (Ashcroft et al., 2021; Camacho et al., 2021; Vargas et al., 2021; Villasenor et al., 2021). UREs have been effective in retaining undergraduates in STEM through their graduate degrees and careers, especially students from historically minoritized communities (Eagan et al., 2013; Hernandez et al., 2018). UREs have been found to positively impact historically minoritized students’ science identity and self-efficacy, especially when they receive quality mentorship (Estrada et al., 2018; Hernandez et al., 2018). Therefore, building a culture where supportive mentors in UREs can operate critically-oriented research communities can increase the representation of URG students in STEM fields (Malcom & Malcom-Piqueux, 2013; Saetermoe et al., 2017). Purposeful diversification efforts, critical mentorship, and justice-based UREs must be strategically combined if URG students are to be retained in all academic and professional STEM contexts (Estrada et al., 2018; Tsui, 2007). Effective mentoring practices grounded in communication, flexibility, trust, and humility – especially when enacted by URG faculty – improve the chances of academic and professional success among URG students (Hund et al., 2018; Romney & Grosovsky, 2023). Using a critical race theory (CRT) lens, we examined the effects of critical mentorship on undergraduate student outcomes in two samples of students: students who were in a URE intervention program and their non-program counterparts. Our goal in this study is to identify the ways critical mentoring in a URE can impact students’ perceived mentor satisfaction, science identity, and science self-efficacy.

### A Critical Race Theory Perspective: Local and Global Relevance

Critical race theory perspectives provide a contrast to traditional pedagogy, providing a culturally responsive paradigm for drawing URG students into STEM fields. CRT education scholars have investigated how race or racism has, for example, influenced a student’s science identity (Chang et al., 2011; Rainey et al., 2018), hindered the integration of faculty of color in academia (Beard & Julion, 2016; Villalpando & Delgado Bernal, 2002), and impacted the transfer culture (Jain et al., 2020; Perez & Ceja, 2010). CRT scholars in education challenge the traditional ideology in higher education and disclose how self-interest, power, and privilege are deeply entrenched in various aspects of the educational system (Baber, 2015; Bimper & Harrison, 2017; Iverson, 2007), creating barriers for students and faculty of color.

Critical race theory in the U.S. was initially applied to legal settings (Bell, 1980) and then became a part of other disciplines including education (e.g., Solorzano, 1997). Noting the localized legal origins of CRT, legal scholars abroad have critiqued the U.S.-centric nature of the framework (e.g., Andrews, 2000). Still, these scholars have also applied CRT to critique their own legal systems in ways akin to the original critical race theorists (e.g., Mutua, 2000). Much like in the U.S., the CRT work of legal scholars abroad has influenced domains outside law (Hylton et al., 2011). This work has highlighted how the intersections of *space*—the physical geography of a locale—and *place*—the historical meaning and cultural identity of a locale—(re)produce racism within ethnically/racially diverse locales and across the globe more broadly (e.g., Aoki, 2000). Space and place, or *geocultural context*, drive unique forms of racial domination/subordination in nations other than the U.S.; CRT offers a framework by which to critique the manifold forms of racism that permeate into all facets of social life, which includes education (see Breen, 2018; Moffitt et al., 2019; Thomas, 2012; Wischmann, 2018). From this standpoint, CRT has demonstrated its robustness, global relevancy, and utility in confronting issues of educational inequity and uncritical mentorship in particular.

### Defining Critical Mentorship

Mentors can cultivate a disposition and practice of inquiry, guide discussions of problems and investigations, interpret and explain observations, and provide a dynamic set of technical as well as psychosocial support (Haverly & Brown, 2022; National Research Council, 2012). However, scientific communities and institutions of higher learning are not immune from the pervasive effects of historical and structural racism. Often, faculty mentors are members of dominant group. Power imbalances between mentors and URG protégés and lack of mentor training obstruct the information of communication and relational trust, which may explain why students of color have reported that white mentors are less helpful than mentors of color (Hansman, 2002).

Much of the traditional (Westernized) mentorship paradigm rests upon a functionalist model whereby mentors find it normal to form hierarchical, unilateral, and race-neutral relationships with so-called “mentees” grounded in individualist, meritocratic, racist, and Western-based ideologies, myths, and discourses (see Augoustinos & Every, 2007, 2010; Augoustinos et al., 2005; van Louw & Waghid, 2008). Mentors come to view mentees as empty receptacles who must be taught the norms and priorities of the already-powerful and oppressive social agents and entities within the STEM community.

Critical mentoring is grounded in social-political principles like race consciousness, a social justice orientation, valuing experiential knowledge, the notion of co-learning, a respect for subjective experience, and the critical analysis of power relations (van Louw & Waghid, 2008). Mentors who are culturally responsive, establish rapport and deep relationships, and discuss matters around diversity are able to create learning contexts wherein URG students experience belongingness, which shape students’ multiple identities (Sotardi et al., 2022). In egalitarian relationships, critical mentors and their protégés may work together to stay current with the latest research, co-construct scientific knowledge, and may even work to bring about positive reforms in the STEM community (Bradbury, 2010).

Via the elevation of numerous voices, the continual negotiation of egalitarian relationships, and openness to the multiplicity of disciplinary knowledge, critical mentors are able to perceive their protégés as burgeoning professionals who, through collaboration, will transform the actions and impacts of an uncritical and socially/politically disconnected STEM community.

Critical race and antiracist approaches have been found to enrich mentored experiences and promote academic, professional, and personal success among URG students (Brown, 2009; Martinez et al., 2018; Quigley & Mitchell, 2018; Saetermoe et al., 2017). Critical mentorship can remedy URG students’ racial stress, system-justifying tendencies, and sense of imposterism (Ashcroft et al., 2021; Camacho et al., 2021; Vargas et al., 2021; Villasenor et al., 2021). In the process, URG students gain self-confidence, enhance their *concientizacion* (i.e., critical social-political consciousness), and are better positioned with justice-oriented scientific research.

Mentors play a critical role in providing not only scientific training, feeding into URG students’ science self-efficacy, but also in supporting their protégé to see oneself as a credible scientist whose innovations are taken seriously – a science identity (Maton et al., 2016; Pajares, 1996; Zheng et al., 2024). Facing stereotypes and stereotype threat throughout their K-12 educational experiences, URG students in STEM require instrumental and socioemotional mentoring (Robnett et al., 2018), community (Maton et al., 2016), an outlet for altruistic goals and recognition of one’s accomplishments (Carlone & Johnson, 2007; Zheng et al., 2024). Flowers & Banda (2016) propose that, given URG student histories, establishing a sense of science self-efficacy, a belief that one can be a capable participant in scientific research, is prerequisite to building a science identity and STEM career interest (Maton et al., 2016; Zheng et al., 2024). Negative (Robnett et al., 2018) or traditional mentoring (Carlone & Johnson, 2007) were associated with poor science identification and persistence, reinforcing the need for new models of mentoring URG students.

### The Intervention Context

To increase the representation of URG students in the sciences, the National Institutes of Health (NIH) created the Diversity Program Consortium (DPC), an initiative to implement and evaluate novel interventions at a variety of academic institutions across the country. Part of the DPC, the Building Infrastructure Leading to Diversity (BUILD) initiative was launched in 2014 with a mission to engage undergraduate students from diverse backgrounds in biomedically-related research at 10 Minority Serving Institutions (MSIs) across the nation, each unique and transformative. The BUILD PODER program approaches the DPC’s larger mission by training sophomores, juniors, and seniors from 22 disciplines in undergraduate research through the framework of critical mentorship.

To develop critical mentoring skills, faculty mentors received training to address unconscious bias, white privilege, historical trauma, stereotype threat, microaggressions, and the continual impact of systemic and institutional racism in STEM (see Saetermoe et al., 2017).

All faculty mentors in the intervention program received, at a minimum, 16 h of CRT-informed mentor training to build mentor-protégé relationship including didactic, role-play, and intentional activities that challenge traditional mentoring. Mentors who began Year 1–4 were offered one to three additional years of training. These mentors, some who received four years of training, discussed how CRT works in their laboratory, how racist structure and symbols enter our very thinking based on a history of racism in the U.S., and participated in a visceral experience that included Augusto Boal’s *Theatre of the Oppressed*. Faculty were offered small mentoring stipends, conference travel funds, and opportunities for $50K pilot projects as well as professional development (e.g., grant-writing workshops, writing groups, speakers and workshops around health disparities).

Similar to traditional interventions, students received stipends, research supplies, travel to conferences, and summer research experiences. Unique to the program, the CRT-informed student training addresses: (a) race and racism, (b) community-building, (c) research ethics, (d) professional development, (e) health and well-being, and (f) advanced skills-building through a two- or three-year faculty-mentored research and advanced courses in their disciplines (see Ashcroft et al., 2021; Camacho et al., 2021; Saetermoe et al., 2017; Vargas et al., 2021; Villasenor et al., 2021 for additional information regarding the student training).

This study examined the relationship between students’ perception of race-conscious mentoring and student outcomes in a sample of STEM undergraduate students in an intervention and students not in an intervention. We examined the following research questions:

1. Do higher levels of critical mentoring positively correlate with increased student outcomes, including higher mentor satisfaction, stronger science identity, and improved science self -efficacy?
2. Do students who participate in the intervention exhibit better outcomes (mentor satisfaction, science identity, and science self-efficacy)?
3. Does critical mentoring serve as a mediator in the relationship between intervention participation and student outcomes?

## Method

### Participants

We recruited undergraduate students in STEM disciplines through an invitation to participate in a survey examining the experiences of STEM undergraduate students. Participants included intervention and non-intervention students. Participants completed the Student Annual Follow-up Survey (SAFS), an online survey administered by national BUILD evaluators at the Coordination and Evaluation Center (CEC) housed by a research 1 university in California. In 2020, the year this study was conducted, a total of 1,385 students completed the survey at the local BUILD campus. A comparison group was identified using propensity score matching (PSM) to extract the intervention effects from the confounded effects coming from both the program and other related factors. The point of PSM is that the intervention program advantage might easily stem, not from the relative effectiveness of the program, but from unobserved differences between those who chose to participate in the program and those who chose not to. For example, parents who encouraged their children to participate in the intervention program may care deeply about the quality of their children’s education. These same parents may also have been more likely to try to enhance their children’s skills at home by emphasizing the importance of reading or by checking their children’s homework (Murnane & Willett, 2010). In this study, we used four matching estimators (i.e., formal training or workshop, conducting own research, honors and awards, and conference attendance or presentation) to control for selection bias while producing the PSM scores using SPSS 25.0 (IBM, 2017). After PSM, there were 1,061 students who produced similar propensity scores. Of the 1,061 survey participants, 158 participated in our intervention program and 903 did not. In our study, the sample sizes varied between groups with and without intervention. When sample sizes are unequal, it can potentially impact the assumption of equal variance. To assess this, Dean and Voss (1999) proposed a simple guideline: if the ratio of the larger variance to the smaller variance is less than three, the assumption of equal variance is likely met. In our case, we examined the ratio of variances between the intervention and non-intervention groups, as shown in Table 1. None of the ratios exceeds three, indicating that the assumption of equal variance was not violated.

**Table 1:** The ratio of the larger variance to the smaller variance between intervention and non-intervention

	Larger variance	Smaller variance	Ratio
Mentor satisfaction	1.641	1.107	1.48
Science identity	0.896	0.407	2.20
Science self-efficacy	0.791	0.611	1.29
Critical mentorship	1.390	1.160	1.20

University Institutional Research (IR) data provided students’ demographic information, which included self-identified binary gender, first generation student status, and whether the students belong to a historically underserved racial/ethnic group (URG: e.g., African American/Black, American Indian/Alaskan Native, Latinx, and Native Hawaiian/Pacific Islander). Demographic characteristics of the participants are displayed in Table 2. In our model, we incorporated gender, first generation status, and information related to underrepresented groups (URGs) as covariates. These covariates were included to account for the impact of these demographic variables on student outcomes.

**Table 2:** Demographic characteristics of the participants

	Frequency	Percent
Gender		
Female	641	60.4
Male	359	33.8
missing	61	5.7
First Gen		
Non-First Gen	243	22.9
First Gen	643	60.6
missing	175	16.5
URG		
Non-URG	288	27.1
URG	692	65.2
missing	81	7.6

Gender is self-identified. A first-generation college student is defined as a student whose parent(s)/legal guardian(s) have not completed a bachelor’s degree at a four-year college or university. URG represents historically underserved racial/ethnic group (e.g., African American/Black, American Indian/Alaskan Native, Latinx, and Native Hawaiian/Pacific Islander)

### Instruments

The 2020 Student Annual Follow-up Survey (SAFS) used in this study consisted of 18 Likert scale survey items: five items are related to critical mentorship, four to science identity, six to science self-efficacy, and three to mentor satisfaction. An aspect of critical mentorship was measured using the behavioral subscale of the Cultural Diversity Awareness to Race/Ethnicity (CDA-R/E) Scale: Mentee Version (Byars-Winston & Butz, 2021); the five-point Behaviors subscale ranges from *never* (1) to *all the time* (5) and has shown high internal reliability (i.e., α = 0.88). The subscale asks students to report the extent to which they discuss issues of race/ethnicity with their mentors across different contexts. An aspect of each of science identity and science self-efficacy was measured using the science identity scale and science self-efficacy scale, respectively, conducted by the NIH Diversity Program Consortium (DPC); the five-point science identity scale ranges from *strongly disagree* (1) to *strongly agree* (5) and the five-point science self-efficacy scale ranges from *not at all confident* (1) to *absolutely confident* (5). Science identity is a measure describing the extent to which students conceive of themselves as scientists and science self-efficacy is a measure of students’ confidence in their ability to conduct scientific research. Mentor satisfaction was measured using seven-point scale ranging from *not at all* (1) to *extremely* (7) and it is a measure of students’ satisfaction with their faculty mentors (see Table 3). SAFS is an online survey administered by the Coordination and Evaluation Center (CEC), the evaluators for all BUILD sites, housed at a major Research І university in California. The Institutional Review Board (IRB) at this university approved the surveys and related protocols. While critical mentorship was used as an independent variable, the remaining items (mentor satisfaction, science identity, and science self-efficacy) were used as dependent variables in this study (see Table 3 again). In addition to these independent and dependent variables, intervention participation was used as a between-subjects variable for this analysis. We utilized gender, first generation status, and URGs as covariates. These covariates were employed to account for the impact of these demographic variables on student outcomes.

**Table 3:** Independent and dependent variables, survey items, construct, and scale for this study

Variable	Item	Construct	Scale
Independent variable	1. My mentor created opportunities for me to bring up issues of race/ethnicity as they arose	Critical mentorship	5-point frequency scale
	2. My mentor encouraged me to think about how the research related to my own lived experience		
	3. My mentor was willing to discuss race and ethnicity, even if it may have been uncomfortable for him/her		
	4. My mentor raised the topic race/ethnicity in our research mentoring relationship when it was relevant		
	5. My mentor approached the topic of race/ethnicity with me in a respectful manner		
Dependent variable1	1. How would you rate the overall quality of the mentoring you received from your primary mentor?	Mentor satisfaction	7-point Likert scale
	2. How satisfied are you with the mentoring you are receiving from your primary mentor?		
	3. To what extent do you feel your mentor is meeting your expectations?		
Dependent variable2	1. I have strong sense of belonging to the community of scientists	Science identity	5-point agreement scale
	2. I derive great personal satisfaction from working on a team that is doing important research		
	3. I have come to think of myself as a scientist		
	4. I feel like I belong in the field of science		
Dependent variable3	1. Use technical science skills (use of tools, instruments, and/or techniques)	Science self-efficacy	5-point frequency scale
	2. Generate a research question		
	3. Determine how to collect appropriate data		
	4. Explain the results of a study		
	5. Use scientific literature to guide research		
	6. Integrate results from multiple studies		

### Analysis

#### Survey Item Validation

The Higher Education Research Institute (HERI) established the validity of the latent constructs of science identity and science self-efficacy through the application of item response theory (Sharkness et al., 2010). For the purpose of survey validation in this study, we checked reliability and validity. For reliability, we examined a *Cronbach’s alpha* (α), separation index, and reliability index from item response theory (IRT) using ConQuest 5.18.5 (Adams et al., 2021) and Winsteps 5.2.0 (Linacre, 2022). According to Boone et al. (2014), a *Cronbach’s α* more than 0.7, separation index more than 2, or reliability index more than 0.8 was considered to have strong internal consistency, high separation, or high reliability (see Table 4).

**Table 4:** Reliability indices, means, and standard deviations of dependent and independent variables

Variable	Construct	Cronbach’s α	Separation	Reliability	Mean (SD)	
					non- Intervention	Intervention
Independent variable	Critical mentorship	0.95	1.95	0.79	3.23 (1.20)	3.85 (1.08)
Dependent variable 1	Mentor satisfaction	1.00	2.45	0.86	5.90 (1.28)	6.32 (1.11)
Dependent variable 2	Science identity	0.88	2.64	0.87	2.97 (0.93)	4.04 (0.68)
Dependent variable 3	Science self- efficacy	0.93	3.03	0.90	2.94 (0.90)	3.69 (0.79)

For validity, we conducted a confirmatory factor analysis (CFA) using Mplus 8.8 (Muthén & Muthén, 1998–2022). As seen in Table 5, factor loadings were above the minimum accepted threshold of 0.40 and suggest items loaded onto the same latent construct (Brown, 2006).

**Table 5:** Factor loadings for independent and dependent variables

IV		DV1		DV2		DV3	
Item	Critical mentorship	Item	Mentor satisfaction	Item	Science identity	Item	Science self- efficacy
1	**0.84**	1	**0.92**	1	**0.83**	1	**0.69**
2	**0.63**	2	**0.93**	2	**0.58**	2	**0.78**
3	**0.88**	3	**0.94**	3	**0.86**	3	**0.86**
4	**0.87**			4	**0.86**	4	**0.84**
5	**0.83**					5	**0.83**
						6	**0.82**

Factor loadings are all > 0.40 and in boldface

*IV *Independent variable, *DV* Dependent variable

#### Structural Equation Modeling

We used structural equation modeling (SEM) regression analysis in Mplus 8.8 (Muthén & Muthén, 1998–2022) to answer our research questions. SEM implies a structure for the covariances between the observed variables and allows for investigation of causality and coordination of multiple factors impacting an independent variable. It is the paths between latent variables that form the structural relationships we are interested in. These path coefficients take the form of regression. However, the advantage of SEM over regression and alternative methods includes but is not limited to: estimates of measurement error in all variables, incorporation of both observed and latent variables, and estimation of direct and indirect effects in a single statistical model (Bryne, 2006). For the measurement component in our model, factor loadings were all above the minimum accepted threshold of 0.40, which means the items used to measure the latent construct are measuring what they are supposed to measure. With regard to the indirect effect or mediation, it refers to a situation when the relationship between a predictor variable and an outcome variable can be explained by their relationship to a third variable or the mediator (Field, 2013). For example, given the training students receive in science skills and research ethics during summer research experiences, intervention participation may directly affect student science identity and self-efficacy (i.e., direct effect). Additionally, intervention participation may provide students with greater opportunities to interact with CRT-trained mentors who validate their experiential knowledge and this CRT-mentored research experience may, in turn, increase science identity and self-efficacy (i.e., indirect effect). Fit indices of measurement and structural model are displayed in Table 6.

**Table 6:** Fit indices of measurement and structural model

Model	χ^2^	*df*	CFI	TLI	RMSEA (90% CI)	SRMR
Measurement Model	558.42***	129	0.95	0.95	0.056 (0.051 − 0.061)	0.06
Structural Model	589.10***	185	0.95	0.94	0.050 (0.046 − 0.055)	0.05

χ2 = chi-square test of model fit, *CFI *comparative fit index, *TLI *Tucker-Lewis index, *RMSEA *root-mean square error of approximation, *SRMR *standardized root mean square residual. CFI or TLI close to 1.00 and RMSEA or SRMR close to 0 can be considered as a good model

***p<.001

## Results

### Research Question 1: CRT-Informed Mentoring Predicts Student Outcomes

**Fig. 1:**
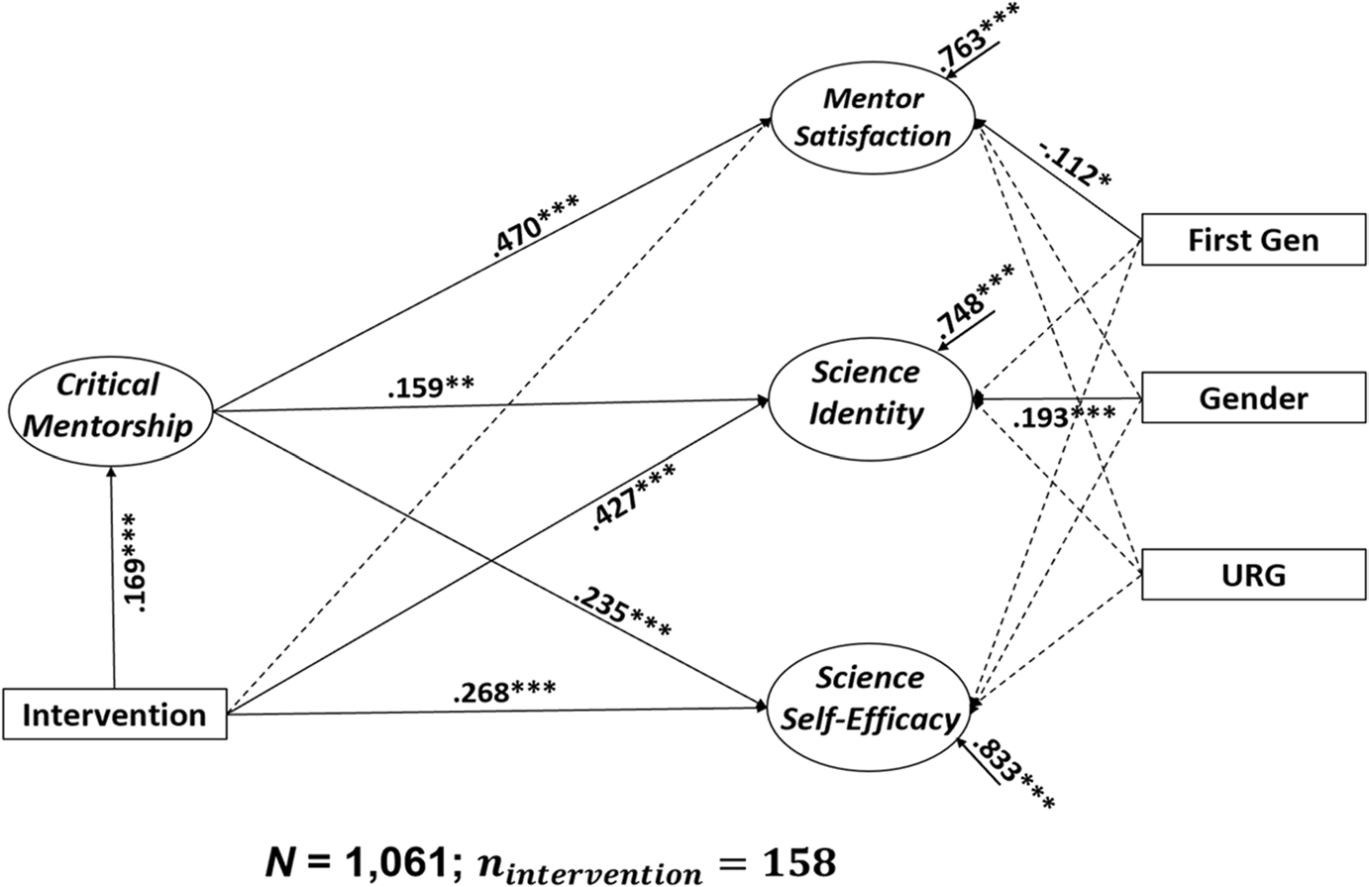
Diagram of structural equation modeling. *Note*. Regression coefficients are all standardized. Ovals represent latent variables. Solid lines indicate a significant relationship and dotted lines represent an insignificant relationship. *p < .05, **p < .01, ***p < .001

As shown in Fig. 1, the results of the SEM – regression showed that CRT-informed mentoring significantly predicted mentor satisfaction, β = 0.470, *p* < .001, science identity, β = 0.159, *p* < .01, and science self-efficacy, β = 0.235, *p* < .001. These results were obtained while accounting for the influence of gender, first generation status, and URGs. The association between CRT-informed mentoring and mentor satisfaction was higher than any other associations.

### Research Question 2: Direct Effect of Intervention Participation on Student Outcomes

The results of the SEM – regression analysis showed a significant direct effect of intervention participation on science identity, β = 0.427, *p* < .001 and science self-efficacy, *β* = 0.268, *p* < .001, while accounting for the influence of gender, first generation status, and URGs. There was not a significant direct effect of intervention participation on mentor satisfaction over and above critical mentorship, β = 0.032, *p* = .420. This implies that mentor satisfaction was derived from critical mentorship, where mentors encouraged students to reflect on how their research connected to their personal life experiences. In contrast, it was not solely based on ordinary mentorship, such as research training or collaborating with faculty mentors during the academic year.

### Research Question 3: Indirect Effect of Intervention Participation on Student Outcomes Mediated by CRT-Informed Mentoring

There was an indirect effect of intervention participation on mentor satisfaction mediated by CRT-informed mentoring, β = 0.169 × 0.470 = 0.079, *p* < .001. This suggests that the intervention program offers participants more extensive opportunities for CRT-informed mentoring compared to students not enrolled in the program. Despite not finding a direct effect of the intervention on mentor satisfaction, these enhanced mentoring opportunities appear to positively impact mentor satisfaction.

In addition, we found another pathway through which intervention participation was associated with higher science identity and science self-efficacy compared to non-intervention participants through CRT-informed mentoring (science identity: β = 0.169 × 0.159 = 0.027, *p* < .01; science self-efficacy: β = 0.169 × 0.235 = 0.040, *p* < .001). These findings imply that the intervention program’s critical mentors provided the context to flourish with respect to meeting mentee expectations (i.e., mentor satisfaction), increasing science skills required to succeed in STEM (i.e., science self-efficacy), and cultivating a sense of belonging to the science community (i.e., science identity).

## Discussion

This study aims to address the significance of critically informed mentoring practices on self-rated student outcomes such as science identity, self-efficacy, and overall mentor satisfaction among intervention participants and a propensity score matched comparison group. In the analysis of SAFS data, a consistent finding emerged: critical mentoring was significantly associated with enhanced science identity, self-efficacy, and mentor satisfaction, irrespective of intervention participation. Interestingly, it appears that non-intervention students may have received mentoring from CRT-trained mentors, albeit outside the formal program structure. We also found that intervention participants reported higher science identity and science self-efficacy than non-intervention participants.

In addition, those in the intervention program reported higher levels of critical mentoring than non-intervention participants which was, in turn, predictive of higher mentorship satisfaction, science identity, and science self-efficacy. This implies that the CRT-informed intervention was able to be more effective by developing an environment in which high-quality, critical mentors influenced students’ sense of science identity and self-efficacy. Nevertheless, the indirect effects of the intervention, which were mediated by critical mentoring, were relatively modest. These effects influenced mentor satisfaction (0.079), science identity (0.027), and science self-efficacy (0.040). In contrast, the direct effects of critical mentoring on these outcomes were more substantial: mentor satisfaction (0.470), science identity (0.159), and science self-efficacy (0.235). Interestingly, the intervention also had direct effects on science identity (0.427) and science self-efficacy (0.268), suggesting that it fostered these aspects through additional pathways beyond critical mentorship. Beyond their direct impact, these indirect effects provide an additional advantage for student outcomes, including mentor satisfaction, science identity, and science self-efficacy. In addition to critical mentorship, the intervention encompasses several supportive components: (a) a four-week intensive training (e.g., mentored research training, CRT and cultural competence in student and faculty professional development, and engaging in team-building activities); (b) working with faculty mentors during the academic year (e.g., conducting research, attending conferences, and disseminating research finding); and (c) social events (e.g., game night, movie night, etc.). These multifaceted activities collectively contribute to students’ growth and development within the program. While the intervention did not directly impact mentor satisfaction, we did observe an indirect effect on this outcome. This suggests that students’ satisfaction with their mentors was influenced by critical mentorship – for instance, mentors encouraging students to connect their research to their personal experiences. In contrast, ordinary mentorship, such as mentored research training or collaborating with faculty during the academic year, did not play as significant a role. The indirect effects of the intervention, mediated by critical mentorship, accounted for a total of 2.13% ($${0.146}^{2})$$ of the variance in student outcomes, including mentor satisfaction, science identity, and science self-efficacy. According to Cohen’s guidelines (2013) for interpreting effect sizes in the social sciences, the indirect effect, accounting for 2.13% of the variance in the model, falls within the range of a small to moderate effect. While modest, this effect holds significance, especially when the research aims to be exploratory or hypothesis-generating (Cohen, 2013). Our study focused on investigating both direct and indirect effects of intervention and critical mentorship on student outcomes.

Findings from this study are aligned with prior research conducted among similar samples looking at the effectiveness of the intervention program. For example, studies have reported the effectiveness of UREs in retaining undergraduates in STEM through graduate degrees and careers, especially students from historically minoritized backgrounds (Eagan Jr. et al., 2013; Hernandez et al., 2018). Studies have also shown UREs to have positively impacted historically minoritized students’ science identity and self-efficacy, especially when they receive quality mentorship (Estrada et al., 2018; Hernandez et al., 2018). To date, no known UREs have incorporated CRT in mentorship except one. The only known study shows that students in intervention receiving CRT mentorship reported significantly greater science personal-identity and significantly great intention to pursue a science career than non-intervention students with a mentor (Ashcroft et al., 2021; Camacho et al., 2021; Vargas et al., 2021; Villasenor et al., 2021). This last study together with the current study underscores the positive impacts of racial consciousness across campus communities and race-conscious practices in mentor-protégé relationships as an effective strategy to improve student science identity, self-efficacy, and overall mentor satisfaction (Norris et al., 2020; Saetermoe et al., 2017).

Critical race theory provides a bridge between universities and faculty mentors and their students of color. When mentors are aware of the history, systems, and interpersonal consequences of racism, they are better able to share a framework and build relationality that invites students of color into biomedical fields.

### Limitations

Four main limitations should be noted when reviewing the results of this study. Firstly, the findings represent data collected from a single Hispanic-Serving Institution (HSI) and thus, may not be generalizable to students and faculty mentors at other institutions who may face varying obstacles such as access, or the lack thereof, to campus-based research opportunities, a hostile institutional climate, and home-school cultural value mismatch (Ashcroft et al., 2021; Guan et al., 2024). Secondly, we did not have specific information about the activities and exposure to resources of non-intervention students or that of their faculty mentors. It may be that non-intervention students were mentored by CRT-trained mentors but not as part of the formal program. Therefore, the possibility that non-intervention students may have had mentors, strengthens the merit of our findings on the effect of intervention participation and critical mentorship. Thirdly, our study did not incorporate qualitative data collection methods, such as interviews. The absence of qualitative approaches may be considered a limitation. Notably, Sablan (2019) delved into the intersection of Critical Race Theory (CRT) and Quantitative Methods (QMs) in her research. In her empirical investigation, Sablan utilized survey data from undergraduates to quantify students’ community cultural wealth. Her work illuminated how quantitative methods can deepen our comprehension of structural racism within educational contexts, effectively bridging the gap between theoretical frameworks and empirical analysis. Nevertheless, it remains essential for researchers to consider both qualitative and quantitative approaches when examining racial disparities in education. Lastly, this study utilized propensity score matching (PSM) and four matching estimators or factors (i.e., formal training or workshop, conducting own research, honors and awards, and conference attendance or presentation) to reduce bias when comparing the intervention and non-intervention groups. PSM creates matched sets of treated (intervention) and untreated (non-intervention) participants who share a similar propensity score. The primary objective of PSM is to balance confounding factors between the intervention and non-intervention groups. By matching participants with similar propensity scores, we aim to make these two groups comparable in terms of these factors. In general, it would be better to use as many factors as possible to ensure that the matching is effective. However, the number of factors should also be limited to avoid overfitting the model. It is important to maintain a balance between these two contradictions. The nature of secondary data limited the number of matching estimators used in the current study, though this study yielded good information which method can be generalized with caution.

## Conclusion

The current study highlights how participation in programs like BUILD can increase science identity and self-efficacy, two factors predictive of science career intentions (Lent et al., 1994, 2008, 2018). The goal of BUILD is to support and encourage students from diverse backgrounds to develop critical and scientific thinking and to pursue and address complex societal health challenges in a science career. This work aligns with the National Academies of Sciences, Engineering, and Medicines (2021) call to action for more equitable science education. The fact that critical mentoring practices are linked to higher science identity and self-efficacy should serve as support to conduct more programs or interventions and continue the momentum.

## Data Availability

All relevant data are within the paper and are available from the corresponding author on reasonable request.
